# Clinical Relevance of the Sympathetic–Vascular Interactions in Health and Disease

**DOI:** 10.3390/biomedicines9081007

**Published:** 2021-08-13

**Authors:** Fosca Quarti-Trevano, Gino Seravalle, Guido Grassi

**Affiliations:** Clinica Medica, Departmenet of Medical Sciences, University Milano-Bicocca, 20126 Milan, Italy; fosca.quarti@unimib.it (F.Q.-T.); g_seravalle@yahoo.com (G.S.)

**Keywords:** sympathetic nervous system, endothelium, cardiovascular disease

## Abstract

The sympathetic nervous system is known to play a pivotal role in the short- and long-term regulation of different cardiovascular functions. In recent decades, increasing evidence has demonstrated that sympathetic neural influences are involved not only in the vasomotor modulation of small resistance arteries but also in the control of large arteries. Sympathetic activity and vascular function, which are key factors in the pathophysiology and prognosis of cardiovascular disease, are linked by a close relationship. Evidence from experimental studies indicates that the sympathetic nervous system is critically influenced, at the central and also at the peripheral level, by the most relevant factors regulating vascular function, namely nitric oxide, reactive oxygen species and endothelin. Additionally, there is evidence of a reciprocal influence between endothelial function and sympathetic mechanisms. This paper will provide an overview of the relationships between endothelial function and the sympathetic nervous system characterizing physiological states. It will also briefly mention the alterations described in cardiovascular disease, with particular emphasis on essential hypertension and congestive heart failure, i.e., the two pathological states in which endothelial dysfunction and neuroadrenergic activation appear to be relevant factors for determining cardiovascular prognosis.

## 1. Introduction

Basic and clinical studies carried out during recent decades have allowed to substantially improve the available information on the role of the sympathetic nervous system in the regulation of cardiovascular function in physiological conditions and in pathologic states directly or indirectly affecting the heart and systemic circulation [1]. Evidence has been provided that sympathetic neural influences, in conjunction with other circulating humoral factors, exert a fundamental role in the homeostatic control of the cardiovascular system [1]. Such interactions represent the key pathophysiological steps in the development and progression of several cardiovascular diseases, becoming important triggers of complications, markers of prognosis and targets of treatment.

The present paper is aimed at providing a critical overview of the clinical relevance of the interactions between the sympathetic nervous system and vascular function in health and disease. We will first examine the neuroadrenergic–vascular interactions, followed by an evaluation of the sympathetic modulation of endothelial function. Then, the relevance of neurohumoral interactions in these complex reciprocal influences will be reviewed. Emphasis will be given to the sympathetic vascular alterations described in cardiovascular and cardiometabolic diseases such as essential hypertension, congestive heart failure, obesity and metabolic syndrome. These conditions share as a common hallmark a hyperadrenergic state and thus may represent models useful for studying the sympathetic–vascular interactions in the presence of a chronic state of sympathetic overdrive. Finally, in the last part of the paper, we will highlight the issues which still remain controversial and thus worthy of future investigations.

## 2. Sympathetic Influences on Vascular Function

The two main adrenergic neurotransmitters, norepinephrine and epinephrine, display approximately similar potencies as agonists of alpha_1_ and alpha_2_ vascular adrenergic receptors, the classical physiological responses mediated by these receptors being vessel smooth muscle contraction resulting in constriction of arterioles and veins [1]. These neurotransmitters participate—along with inflammatory mediators, oxidative stress, metabolic factors and genetic background—in the development and progression of the phenomenon known as “arterial stiffness”, i.e., the rigidity of the arterial wall determined by structural components of the vessel [2,3]. Evidence has been provided that an increase in sympathetic vascular drive may directly or indirectly favor the development and progression of the arterial wall stiffening [4,5]. The process has a solid pathophysiological background based on evidence that sympathetic neural factors exert profound modulation of a major component of arterial stiffening, i.e., an arterial distensibility. Intravenous infusion of phenylephrine, a vasoactive drug able to trigger potent vasoconstriction, is associated with a prompt reduction in radial artery distensibility as assessed by beat-to-beat changes in vessel diameter [6]. A similar marked reduction in radial artery distensibility has been reported in response to the cold pressor test, i.e., a maneuver known to augment blood pressure and heart rate because of a diffuse central and reflex increase in sympathetic cardiovascular drive [7,8]. Finally, a reduction in the radial artery and also in the carotid artery distensibility has been reported during acute cigarette smoking [6], i.e., a behavior that is accompanied by a marked increase in blood pressure and heart rate because of the peripheral (and probably central) sympathostimulating effects of nicotine and other smoking products [9].

Whether the influences sympathetic activity exert on arterial vessels are intermittent (phasic) or continuous (tonic) has been examined in a number of studies [4,5,10,11]. In one study, radial artery distensibility was assessed before and after ipsilateral anesthesia of the brachial plexus in patients prepared for surgical correction of Dupuytren’s disease [12]. The anesthetic procedure did not cause any substantial change in blood pressure and heart rate, but it was accompanied by an increase in arterial distensibility throughout the diastosystolic blood pressure range. Similar results were seen at the level of the femoral artery after ipsilateral subarachnoid anesthesia in healthy subjects undergoing arthroscopic removal of a meniscal lesion [12]. Thus, removal of sympathetic vascular influences is accompanied by an increase in arterial distensibility, which means that the ongoing sympathetic tone exerts a restraining action on this arterial function [12].

Another condition that can physiologically impair the relationships between the sympathetic function and arterial stiffness is represented by the aging process, which is associated with both marked physiological sympathetic stimulation and a reduction in the viscoelastic properties of the large arteries [13]. A further factor that may be relevant in considering the relationships between arterial stiffness and sympathetic activity is related to gender, given the evidence that an inverse relationship between adrenergic drive and augmentation index has been reported in males but not in females [14].

## 3. Mechanisms of the Sympathetic–Vascular Interactions

An increase in sympathetic activity may reduce arterial distensibility via at least two non-mutually exclusive mechanisms. First, distensibility may be reduced because the resulting increase in vessel diameter stretches the less distensible component of the vessel wall (e.g., collagen), making the relationship an inverse one [3,4,5]. Second, distensibility can be reduced because of a sympathetic-dependent increase in heart rate, given that this increase is associated with a stiffening of medium-sized and large elastic arteries in both animals and humans [15,16]. An additional mechanism may consist in contraction of the vascular smooth muscle because the elastic modulus of contracted muscle tissue is greater than that of the relaxed one [12].

## 4. Sympathetic–Endothelial Interactions

**Figure 1:**
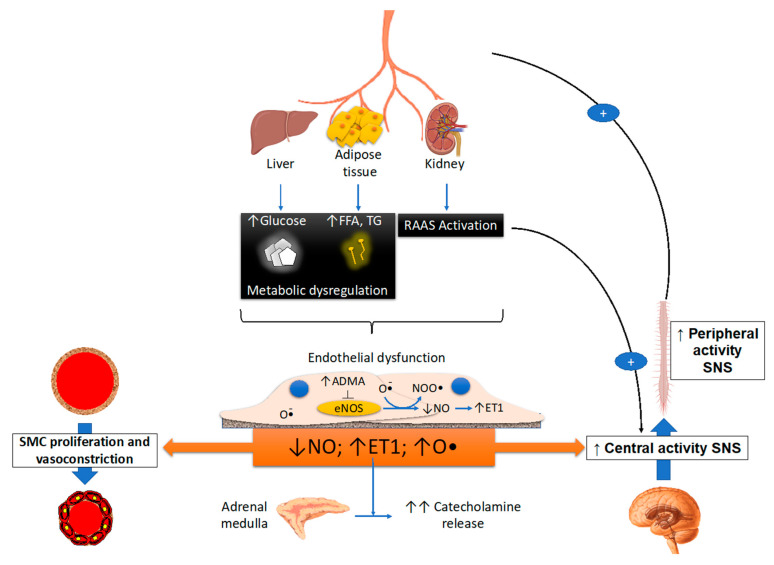
Schematic drawing illustrating the metabolic, endothelial, nitric oxide and vascular smooth cell proliferation of central and peripheral stimulation. ET: endothelin, FFA: free fatty acid, TG: triglycerides, NO: nitric oxide, O: oxide, ADMa: asymmetric dimethylarginine, RAAS: renin-angiotensin system, SMC: smooth cell proliferation, SNS: sympathetic nervous system. The symbol + refers to a stimulating effect, while arrows refer to the possible interactions between various variables.

The endothelium releases a number of factors, some of them with potent vasoconstrictive effects, such as endothelin I and angiotensin II, and some others with known vasorelaxing properties, such as prostacyclin, endothelium-derived hyperpolarizing factor, adenosine, substance P, acetylcholine and nitric oxide [17]. Several lines of evidence have established that nitric oxide, produced from L-arginine by endothelial nitric oxide synthase, is a potent sympathoinhibitory substance acting within the central nervous system [18]. Factors that may trigger nitric oxide production include platelet-derived substances, acetylcholine and cytokines [18]. A consistent component of nitric oxide is generated by phagocytes as a component of the immune response. Nitric oxide synthase activity and its inhibition have been shown to trigger blood pressure increase and concomitant sympathetic activation. Both these responses were potentiated by arterial baroreceptor deafferentation and abolished by cervical spine section, indicating that the monomethyl L-arginine pressor effect is, by and large, mediated by the sympathetic nervous system [18]. Nitric oxide, however, has been shown to exert influences on peripheral sympathetic drive, acting on sympathetic nerves, ganglia and adrenal glands. Two other vasoactive substances known for their interactions with the sympathetic neural function deserve to be mentioned. First, reactive oxygen species, such as superoxides, contribute to oxidative stress, which in turn stimulates central sympathetic outflow in a variety of pathological conditions. Acute correction of oxidative stress with vitamin C or other antioxidants leads to sympathetic inhibition [18]. The interaction between adrenergic function and oxidative stress may occur not only in the brainstem but also at the level of the peripheral nerves. Furthermore, endothelin 1 produced by endothelial cells is a potent vasoconstrictor agent which can stimulate central and peripheral sympathetic drive through endothelin receptors ET_A_ [16,17,18,19] (Figure 1). It should finally be mentioned that in healthy individuals, a significant correlation has been found between muscle sympathetic nerve traffic and surrogate markers of endothelial function [20]. Similar findings have been reported by Lambert and coworkers [21].

Sympathetic/endothelial interactions have also been documented in humans as a result of a variety of acute physiological interventions able to produce a marked enhancement of sympathetic cardiovascular drive, such as a mental arithmetic task, cold pressor test and isometric handgrip exercise [12]. Chronic pathological conditions characterized by a sympathetic overdrive also display profound changes in endothelial function. These will be reviewed in the next paragraph.

## 5. Sympathetic–Vascular Alterations in Cardiovascular Disease

The vast majority of clinical conditions known to be characterized by an adrenergic overdrive, including essential hypertension, congestive heart failure, chronic kidney disease, obesity and metabolic syndrome, share endothelial dysfunction as a common hallmark [12]. It should be emphasized that in some of the above conditions, sympathetic overdrive and, concomitantly, endothelial dysfunction have a clear-cut clinical relevance because both have been shown to represent independent markers of the disease prognosis [22,23,24,25,26].

The increased sympathetic activity reported in clinical conditions characterized by high blood pressure appears to be a peculiarity of the essential hypertensive state, being undetectable in the secondary forms of the disease [27]. Furthermore, various studies have convincingly shown that the magnitude of the adrenergic overdrive parallels the severity of the hypertensive state, becoming more and more elevated as the clinic or ambulatory blood pressure values progressively increase [27]. Although several studies support the “central neurogenic “ nature of the hypertension-related sympathetic overdrive, evidence has been provided showing that a decrease in nitric oxide levels can “per se” at least in part be responsible for the augmented sympathetic neural drive [28,29]. Similarly, in patients with chronic kidney disease, also characterized by a marked sympathetic activation [23], circulating plasma levels of asymmetric dimethylarginine, an inhibitor of nitric oxide, are elevated [30].

**Figure 2:**
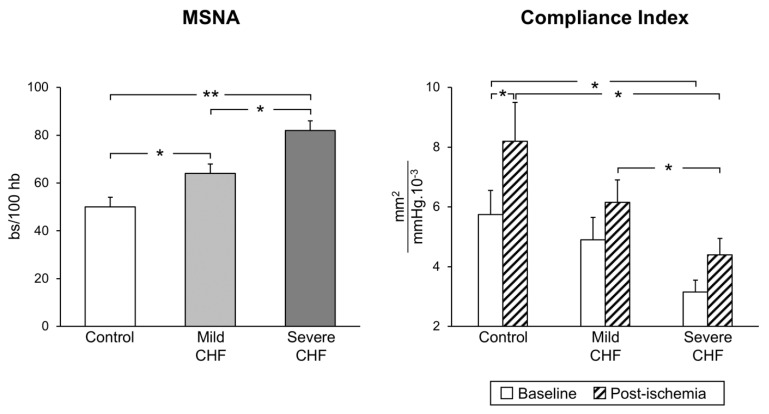
Progressive increase in sympathetic nerve traffic (left panel, MSNA, as assessed via microneurography) and progressive decrease in arterial distensibility (compliance index, right panel) from the healthy state (control subjects) to patients with mild and severe congestive heart failure (CHF). Bs: bursts; HB: heart beats. Asterisks (* *p* < 0.05, ** *p* < 0.01) refer to the statistical significance between groups. From data presented in Ref. [6].

The situation appears to be more complex in congestive heart failure, which is characterized by functional and structural cardiovascular alterations which involve the cardiac pump and the peripheral circulation as well. The latter include, among others, a reduction in arterial distensibility and, thus, an increase in arterial stiffness, which may favor the progression of the heart failure state by determining changes in central hemodynamics [31]. A number of factors, alone or in combination, participate in the development and progression of the arterial stiffness alterations, including sympathetic activation [32]. It is worth mentioning that, firstly, a significant relationship exists between the reduction in arterial distensibility (and thus the increase in arterial stiffness) and the magnitude of the adrenergic overdrive, and secondly, cardiac sympathetic activation appears to be closely linked to pulmonary arterial pressure, independently of heart failure etiology [33]. Regarding the mechanisms responsible for the neuroadrenergic overdrive characterizing heart failure, growing evidence supports the notion that the central sympathoinhibitory effects exerted by nitric oxide appear to be impaired in this condition, presumably because of a decrease in the availability of neuronal nitric oxide synthase [34]. This represents the pathophysiological background for the finding that endothelial dysfunction appears to be a hallmark of the heart failure state. In this context, it is worth mentioning that both endothelial (and more generally vascular) alterations and adrenergic overdrive share a common behavior. Namely, the alterations occur early in the clinical course of the disease, being detectable in a mild heart failure state (patients belonging to New York Heart Association functional classes I and II) and becoming more and more pronounced as the clinical severity of the disease increases (Figure 2) [6].

An intriguing question is whether and to what extent the sympathetic abnormalities described in hypertension and in congestive heart failure can be reversed by drug treatment. Evidence has been provided that different pharmacological interventions may favor a partial restoration (although not a full normalization) of the sympathetic abnormalities described in the abovementioned clinical conditions [32,35]. This may particularly be the case for drugs, such as angiotensin-converting enzyme inhibitors, angiotensin II receptor blockers and, among beta blockers, nebivolol, which combine the sympathomoderating effects with a favorable action on nitric oxide.

Cardiometabolic diseases characterized by a marked sympathetic activation, such as obesity and metabolic syndrome [36], also display profound alterations in the structural and functional characteristics of large and medium arteries. These abnormalities, which include an increase in arterial stiffness, a reduction in arterial distensibility and an impairment of endothelial function, have also been reported in small resistance arteries [36,37]. Given the close relationship found between the functional and structural alterations described in the coronary and subcutaneous small arteries, these vascular and endothelial abnormalities may have important implications for the progression of organ damage related to cardiovascular risk factors and for determining cardiovascular prognosis [38].

## 6. Concluding Remarks

Despite the remarkable achievements in understanding the relevance of sympathetic–vascular interactions in the clinical arena, a number of issues remain still controversial, which will be targets of future investigations. These include the potential relevance of the genetic background in the modulation of endothelial and sympathetic functions and their interactions [39]. They will also include the participation of immune mechanisms in the vascular–sympathetic crosstalk [40]. A further area of future research is represented by an in-depth investigation on how the sympathetic–vascular interactions may participate in determining the so-called “residual cardiovascular risk”, i.e., the risk of incidence of vascular events or progression of established vascular damage persisting in patients despite treatment with current evidence-based recommended care [1,27]. Among the various factors responsible for the phenomenon, a likely one is represented by the finding that pharmacological interventions, although able to correct the sympathetic and endothelial abnormalities reported in cardiovascular disease, are unable to achieve complete normalization of the neuroadrenergic–vascular functions, with adverse consequences for cardiovascular health [41]. This conclusion may be extended to some of the cardiovascular-device-based interventions recently introduced for the treatment of cardiovascular disease (hypertension and heart failure in particular), such as renal nerve ablation and carotid baroreceptor stimulation, which have been shown to exert not always univocal effects on sympathetic and vascular endothelial function [23,29]. Future studies are thus needed also in this specific new area of cardiovascular therapeutic intervention.
